# Understanding the impact of sisu on workforce and well-being: A machine learning-based analysis

**DOI:** 10.1016/j.heliyon.2024.e24148

**Published:** 2024-01-07

**Authors:** Umair Ali Khan, Janne Kauttonen, Pentti Henttonen, Ilmari Määttänen

**Affiliations:** a Haaga-Helia University of Applied Sciences, Helsinki, Finland; b University of Helsinki, Helsinki, Finland

**Keywords:** Sisu, Mental resilience, Work-life satisfaction, Mental wellbeing, Work performance, Work efficiency, Machine learning

## Abstract

This study investigates the construct of sisu, a Finnish attribute representing mental resilience and fortitude when confronted with difficult situations. By leveraging advanced analytical methods and explainable Artificial Intelligence, we gain insights into how sisu factors influence well-being, work efficiency, and overall health. We investigate how the beneficial aspects of sisu contribute significantly to mental and physical health, satisfaction, and professional accomplishments. Conversely, we analyze the harmful sisu and its adverse impacts on the same domains. Our findings, including intriguing trends related to age, educational level, emotional states, and gender, pave the way for developing tailored solutions and initiatives to nurture the beneficial aspects of sisu and curtail the damaging consequences of sisu within professional settings and personal welfare.

## 1 Introduction

This study concerns the Finnish concept of sisu, which represents resilience and inner strength against challenges. Sisu refers to extraordinary determination, courage, and firmness in the face of adversity. It is about having the mental toughness to continue to fight against a difficult challenge, not just to survive, but to overcome. An individual might show sisu by continuing to work towards important goals despite hindrances such as emotional setbacks, recovering from a serious injury or illness, or building a business even after facing repeated failures. It is important to highlight that sisu is typically a Finnish concept, with no direct analogs in other languages or cultures.

While sisu is recognized for its positive impact, its scientific exploration is relatively recent. The most significant difference between the notion and concept of sisu and pre-existing mental fortitude scales such as resilience scale [1], mental toughness scale [2], hardiness scale [3], self-efficacy scale [4], hope scale [5], and grit scale [6] is that the definition of sisu includes both beneficial and harmful sides. Our previous work on sisu [7,8] has revealed a reciprocal relationship between sisu and various psychological and work-related aspects, including well-being, work stress, and drop-out tendencies. We have shown that favorable aspects of sisu correlate with positive outcomes while its adverse elements lead to negative consequences. Specifically, harmful sisu can intensify work stress and aligns with the effort factor in Siegrist's work stress model [9]. These findings suggest that individuals with higher levels of harmful sisu may face challenges in effectively directing their efforts at work. Our research sheds light on the complex interplay of sisu in both personal and professional contexts.

In our prior work [7], we formulated and validated a questionnaire, the Sisu Scale, that effectively measures both beneficial and harmful sisu. In the current study, we supplement it by formulating several questions that include categorical options related to demography, perception of health, and working environment, as well as various open-text questions (see Section 2). Utilizing the Sisu Scale developed in Ref. [7], we calculate six sisu factors (3 beneficial, 3 harmful) for each respondent based on the collected data which collectively show the mental resilience of an individual.

In the present study, we explore sisu deeper under the powerful lens of machine learning to get better insights into the underlying relationships between the novel survey variables and the sisu factors. Since sisu has been shown to be a reliable predictor of well-being when assessed through self-reported Likert scales, we aim to investigate how personal perceptions conveyed in terms of open-text responses interact with sisu and overall well-being. This study seeks to answer two important questions: Are the responses to the open-text questions, representing the overall feeling and mental resilience, linked to higher well-being? And can sisu, as measured by the Sisu Scale, predict positive and negative well-being based on these textual responses? By addressing these inquiries, we aim to shed light on the influence of sisu on individual and societal well-being.

We employ Explainable AI (XAI) methods to understand the relationships, roles, and influences of various predictors on sisu factors and the subsequent impact of these sub-factors on well-being, linguistic variables, work performance, and health status. For instance, a manual analysis of the collected data may not reveal the precise way sisu factors affect health status or work performance. Certain sisu factors might not play a significant role in determining health status, whereas others could be dominant in predicting it. Utilizing XAI allows us to uncover the underlying relationships between these variables and obtain insights into the prediction mechanisms at play.

By applying an XAI framework, we aim to identify the factors contributing to fortitude and their influence on physical and mental well-being, satisfaction, health status, overall feelings, and work performance. Simultaneously, we develop a more profound understanding of the interactions between sisu elements and diverse predictors. This knowledge aids in formulating focused initiatives and tactics. To the best of our knowledge, the interplay between sisu and various other factors such as demography, health, work life, and personal perspective factors has not been investigated with machine learning, which could unveil the relationships and patterns that traditional statistical methods might overlook. By leveraging machine learning, we may uncover subtle interactions between these variables, thereby providing a more comprehensive understanding of how sisu influences individuals in their professional and personal lives. Our findings can contribute to the design of more effective interventions and strategies for promoting beneficial sisu and mitigating the harmful effects of sisu in the workplace and individual well-being. Ultimately, this analysis will aid in fostering resilience and determination in the face of adversity while minimizing the negative consequences of sisu on employees' health and productivity.

The remainder of this paper is structured as follows. Section 2 presents an overview of the related work in this domain. In Section 3, we present an in-depth examination of the used dataset, offering insights from both a data analysis and machine learning lens. Following that, Section 4 elaborates on the preprocessing steps employed in our study. It outlines the use of K-means clustering to optimally cluster textual responses, the annotation of these responses, the evaluation of inter-rater agreement, and the methods used to estimate missing values. Moving forward to Section 5, we analyze sisu data employing gradient boosting. We detail both the regression and classification analysis processes, including hyperparameter tuning. Next, Section 6 presents a comprehensive report of the results obtained from the regression and classification analysis. In Section 7, we offer a broad discussion that synthesizes our findings, helping to articulate the implications of our work. We wrap up the paper in Section 8, reflecting on our conclusions and offering a glimpse into potential future directions.

## 2 Related work

The exploration of machine learning techniques to enhance work-life balance and overall quality of life has been a focus of recent research. For instance, a study employed a machine learning tool to identify factors influencing work-life balance using a dataset from 800 employees [10]. This study identified key elements such as working hours, free time, and financial status that influenced work-life balance. Furthermore, the tool was effective in predicting shifts from balance to imbalance and vice versa. The relevance of machine learning techniques in quality of life assessments was also demonstrated in a study that applied machine learning algorithms to the World Happiness Report data [11]. The study found that regression algorithms performed well in predicting the quality-of-life indicator for the year 2021. In a similar vein, researchers used machine learning techniques to identify the impact of work environment factors on mental health. This focus was seen in a study examining work environment predictors of mental health in nurses [12]. Other studies have also been conducted to understand the influence of various psychosocial factors on the quality of life and well-being among older adults using machine learning techniques [13]. Additionally, the exploration of mental health in the workplace using machine learning techniques has been another focal point in research. The data from a health survey was analyzed to understand the factors influencing mental health disorders among employees [14]. The main goal was to raise public awareness about mental illness in the workplace.

The onset of the COVID-19 pandemic has further emphasized the importance of machine learning techniques in mental health research. Research using machine learning algorithms to assess mental well-being based on a survey from 17 Southeast Asian universities showed that random forest and adaptive boosting algorithms were most successful in identifying negative mental well-being traits [15]. The study identified sports activities, body mass index, grade point average, sedentary hours, and age as key predictors. Similarly, another research [16] integrated demographic variables (such as age and gender) along with certain lifestyle habits and well-being practices (ranging from diet and physical activity to sleep and smoking habits) in their predictive model for mental well-being. Using a gradient-boosting approach, a more recent approach [17] utilizes wearable sensors to measure heart rate variability to successfully predict a person's degree of resilience, optimism, and emotional support.

While these studies have demonstrated the potential of machine learning in evaluating mental health, they have only touched upon a fraction of the factors involved in mental resilience. In particular, they do not consider a wide range of factors in combination such as demography and employment, work environment conditions, well-being practices, and personal perspectives. Neither do they address the harmful and beneficial sisu factors which are the focus of this study. Furthermore, the concept of sisu, with its holistic approach encompassing mental well-being, work environment satisfaction, and work-life balance, remains unexplored within the realm of machine learning. In contrast, our research explores a wide range of influencing factors that collectively contribute to sisu.

## 3 Materials

**Fig. 1:**
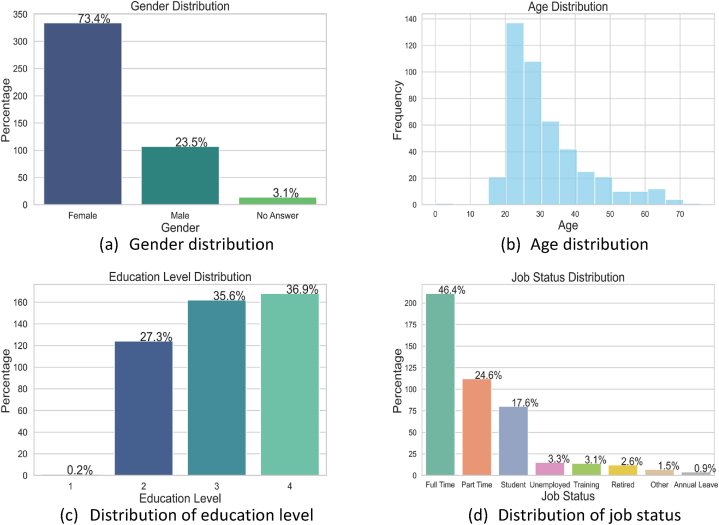
Demographic distribution of the survey participants according to different factors.

The data presented was garnered from the “Sisu in Working Life” project (2022), funded by the European Social Fund Plus. There were 455 participants with age range from 19 to 76. Most of the participants (n=211) were full-time workers during the data collection phase. Notably, a significant portion were part-time students (n=117), a demographic we accessed through university mailing lists. The dataset consisted of 46.37 % full-time workers, 25.71 % part-time workers, 27.92 % other nature of job, and 73.41 % females with an average age of 31.95 years. Fig. 1 shows the demographic distribution of the survey participants according to different factors. The third figure shows the educational levels of the participants grouped into 4 levels where 1 represents the primary and lower secondary education (lowest level) and 4 represents the highest level (university education).

Our online survey^1^ incorporated the Sisu Scale introduced in Ref. [7]. Both the six sub-factors and the two primary factors of the 16-item Sisu Scale were determined following the guidelines set out in Ref. [7]. Additionally, work-related stress was assessed using Siegrist's scale [18], while well-being was calculated through the WHO5 instrument [19]. The survey further collected demographic data, insights into health perceptions, inquiries about the work environment, and also featured open-ended questions for participants.

**Fig. 2:**
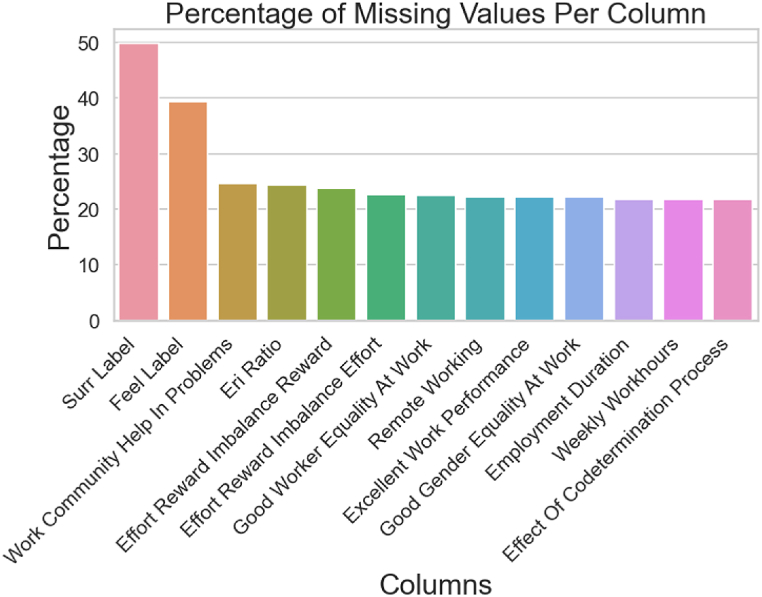
Variables with missing values.

The dataset comprises 41 columns, with 35 of them representing distinct variables and the remaining 6 dedicated to sisu sub-factors. With 455 samples (rows), the dataset contains a variety of data formats, such as numerical, categorical, and text. As the data originates from a survey, many null values are present, corresponding to participants' blank responses. Fig. 2 illustrates the proportion of null values in columns containing missing data. Approximately 37 % of the columns exhibit null values accounting for up to 50 % of their content, posing a challenge for training a model that delivers satisfactory performance. Consequently, addressing missing values is a crucial step in this machine learning-based analysis to ensure robust and accurate results.

The six sisu sub-factors comprise three beneficial and three harmful sisu factors. The three beneficial sisu sub-factors include latent power, extraordinary perseverance, and action mindset. The three harmful sisu sub-factors include harm-to-self, harm-to-rationality, and harm-to-others. Detailed explanation of all sub-factors is given in Ref. [7].

The variables in sisu dataset are divided into four categories:

1. **Demographics and Employment:** This group encompasses variables related to an individual's background, personal demographics, and employment status. It includes information about age, gender, education level, and job situation, such as full-time or part-time employment, training, annual leave, layoffs, unemployment, retirement, student status, and other job types. Additional variables in this cluster cover work position, employment duration, and weekly work hours, providing a comprehensive overview of the individual's work and demographic profile.
2. **Work Environment and Conditions:** This group centers on elements of the work setting and conditions that impact an employee's experience on the job. Aspects such as the influence of collaborative decision-making, problem-solving support from colleagues, remote work arrangements, and perceptions of exceptional job performance are considered. Additionally, the group underlines the significance of workplace equality, incorporating variables measuring strong gender and employee fairness, as well as career advancement opportunities. Lastly, it includes variables pertaining to imbalances between effort and reward, addressing both the reward and effort components.
3. **Well-being and Health:** This group comprises variables measuring an individual's overall well-being, mental and physical health, and life satisfaction. It includes variables indicating the individual's stress levels, financial stress, overall life satisfaction, health status, and sleeping problems. The cluster also covers physical exercise habits and concerns about external factors, such as worries related to Ukraine. Additionally, it includes variables related to effort-reward imbalance ratio (ERI_RATIO) and the World Health Organization's well-being index (WHO5).
4. **Personal Perspectives:** The variables in this group represent textual responses from individuals, offering insights into their subjective experiences and emotions. The surrender variable captures an individual's breaking point, i.e., the moment they think they would surrender to a difficult situation; whereas, the feel variable reveals their overall feelings (good or bad) about their work life. This cluster provides a more personal, qualitative perspective on the individual's experiences and emotions. All responses except one were given in Finnish.

**Fig. 3:**
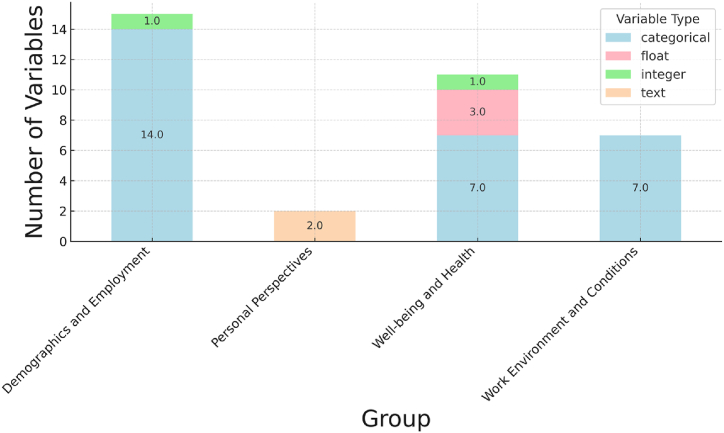
Number and type of variables in each group.

Fig. 3 shows the number and type of variables in each group. It is important to differentiate between categorical and integer variables, even though they may share the same data format. This is because the range of categorical variables is typically much smaller than that of integer variables. For example, while an integer variable like age can take on a range of values from 22 to 76, a categorical variable like health status may only have 5 values ranging from 1 to 5. Therefore, it is important to correctly classify variables as either categorical or integer based on their range of values. Table 1 shows the variables in each group and their type and range.

**Table 1:** Variables in each group and their type and range.

Group	Variable	Type	Range
**Demographics and Employment**	Gender	categorical	1–2
	Age	integer	20–68
	Education level	categorical	1–4
	Job fulltime	categorical	0–1
	Job parttime	categorical	0–1
	Job training	categorical	0–1
	Job annual leave	categorical	0–1
	Job laid off	categorical	0–1
	Job unemployed	categorical	0–1
	Job retired	categorical	0–1
	Job student	categorical	0–1
	Job other	categorical	0–1
	Work position	categorical	1–6
	Employment duration	categorical	1–5
	Weekly workhours	categorical	1–5
**Work Environment and Conditions**	Effect of codetermination process	categorical	1–3
	Work community help in problems	categorical	1–3
	Remote working	categorical	1–4
	Excellent work performance	categorical	1–6
	Good gender equality at work	categorical	1–6
	Good worker equality at work	categorical	1–6
	Employment prospects	categorical	1–5
**Well-being and Health**	Feeling stressed	categorical	1–5
	Financial stress	categorical	1–5
	Overall life satisfaction	categorical	1–5
	Health status	categorical	1–5
	Sleeping problems	categorical	1–4
	Physical exercise	categorical	1–4
	Ukraine worries	categorical	0–4
	Effort reward imbalance reward	float	22.0–54.0
	Effort reward imbalance effort	float	6.0–25.0
	ERI RATIO	float	0.26–2.3
	WHO5	integer	16–100
**Personal Perspectives**	surrender	text	n/a
	feelings	text	n/a

## 4 Data preparations

In this section, we outline the measures employed to prepare the sisu data for subsequent examination. We specifically use K-means clustering on the personal perspective group to determine the number of clusters present in each text variable within the group. We then transform these text variables into categorical variables based on the identified clusters and allocate binary labels to each data point. We compute inter-rater agreement to verify the dependability of the labels assigned by the raters. Moreover, we train two language models to further validate the quality of the annotations. Lastly, we estimate missing values in the dataset to guarantee that the data is complete and prepared for additional analysis.

### 4.1 K-means clustering

In this section, we discuss our approach to clustering the “feel” and “surrender” variables in the “Personal Perspectives” group, which contain a diverse range of participants’ textual responses that cannot be directly used in data analysis. To overcome this challenge, we use the K-means clustering algorithm [20] to find the optimal number of clusters for both variables.

**Fig. 4:**
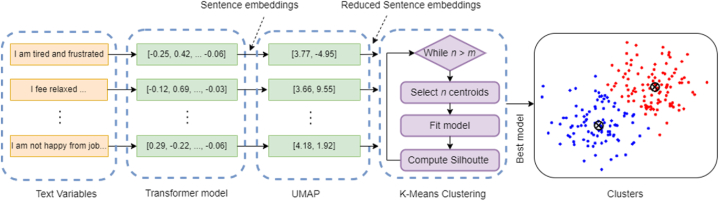
Clustering of sentence embeddings for personal perspectives using the K-means algorithm.

To prepare the data for clustering, we first convert the text observations into numerical data using a pre-trained sentence-transformer model [21] that can derive semantically meaningful sentence embeddings. We apply Uniform Manifold Approximation and Projection for Dimension Reduction (UMAP) [22] to reduce the dimensionality of the sentence embeddings and their impact on distance measures in clustering [23]. Subsequently, we train a K-means clustering model on the truncated data. K-means is an unsupervised clustering algorithm that groups data items into a specified number of clusters. It starts by assigning random positions (centroids) to the clusters and then groups the points in the nearest cluster. It then iteratively finds the average position of the points and moves the respective centroid to that position until all points are at the minimum distance from their respective centroids. Since K-means clustering only groups the data items in a specified number of clusters and cannot find the optimal number of clusters prior to grouping, we generate multiple clustering models and evaluate their performance using the silhouette score [24]. The silhouette score is computed using the mean intra-cluster distance and the mean nearest cluster distance for each sample, and a high score indicates that the clusters are well separated and distinguishable. We select the model with the highest silhouette score, which represents the optimal number of clusters. The process is illustrated in Fig. 4.

We find K-mean clustering to be a better choice for this task. Other clustering algorithms such as Density-Based Spatial Clustering of Applications with Noise (DBSCAN) and Hierarchical Density-Based Spatial Clustering of Applications with Noise (HDBSCAN) are highly sensitive to parameter settings (e.g., distance threshold, and minimum number of points in a dense region). Therefore, we stick to K-means for its simplicity and interpretability. Also, K-means, due to being a centroid-based algorithm, is more useful for summarizing or understanding the common themes or sentiments of the textual responses within each cluster.

### 4.2 Annotation

After determining the optimal number of clusters from K-Means clustering, we assign each text variable with an equal number of labels. For both “feel” and “surrender” variables, we identified two clusters. To facilitate data analysis, we manually labeled the responses and transformed them into categorical variables. Each variable was then categorized into two classes.

Positive (1) Sentiments for “feel” variable:

• Expressions of contentment, happiness, satisfaction, or joy
• Positive language, such as “*I love my job*” or “*I feel fulfilled.*"
• Descriptions of positive experiences, such as “*I get along well with my colleagues*” or “*I feel appreciated by my boss.*"

Negative (0) Sentiments for “feel” variable:

• Expressions of disappointment, frustration, or dissatisfaction
• Negative language, such as “*I hate my job*” or “*I feel drained.*"
• Descriptions of negative experiences, such as “*I don't get along with my colleagues*” or “*I feel undervalued by my boss.*"

Positive (1) Responses for “surrender” variable:

• Clear and specific plans for dealing with a surrendering situation.
• Demonstrated confidence in handling difficult situations.
• Expressions of determination or resilience, such as “*I would never give up*” or “*I am a fighter.*"
• An attempt to answer the question by a detailed text, rather than giving up.

Negative (0) Responses for “surrender” variable:

• Unclear or vague responses that do not address the question directly.
• Expressions of confusion or lack of understanding the question
• Demonstrations of defeat or giving up, such as “*I would give up easily*” or “I am not sure how to handle difficult situations."

**Fig. 5:**
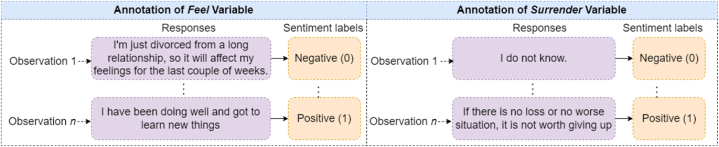
Annotating sentiments for *feel* and *surrender* variables.

The annotation process for feel and surrender responses is illustrated in Fig. 5.

### 4.3 Finding inter-rater agreement

Three annotators (the first and second co-authors, and a research assistant) perform the annotations for the variables “feel” and “surrender” in the dataset. We further evaluate the inter-rater consistency between the annotators. To determine the agreement among the annotators, we utilize Cohen's Kappa coefficient [25], binomial test, and McNemar test which are effective measures of inter-rater consistency.

The calculation of Cohen's Kappa (κ) is described by Eq. (1).

(1) $$\kappa =\frac{\left(P_{o}-P_{e}\right)}{\left(1-P_{e}\right)}$$

Where $P_{o}$ represents the observed agreement between raters and $P_{e}$ represents the expected agreement under the assumption of independence. A higher Kappa value indicates better agreement between raters, with values typically ranging from 0 (no agreement) to 1 (perfect agreement). The binomial test is used to assess the significance of the observed agreement, given a hypothesized probability of success (e.g., p=0.5). The p-value of the binomial test ($P_{binomial}$) is described by Eq. (2).

(2) $$p_{binomial=\sum_{k=0}^{n}C\left(n,k\right)p^{k}{\left(1-p\right)}^{\left(n-k\right)}}$$

where n is the total number of trials, k is the number of observed successes, $p_{binomial}$ is the hypothesized probability of success, and c(n,k) is the number of combinations of n items taken k at a time. A low p -value indicates that the observed agreement is significantly different from what is expected by chance. Lastly, McNemar's test is employed to compare the marginal homogeneity of the two raters. It is based on a chi-square statistic (${\;\chi }^{2}$), given by Eq. (3).

(3) $${\;\chi }^{2}=\frac{{\left(\left|b-c\right|-1\right)}^{2}}{\left(b+c\right)}$$

where b and c are the off-diagonal elements of a 2×2 contingency table containing the counts of agreements and disagreements between annotators. The p -value for McNemar's test is then derived from the chi-square distribution with 1 degree of freedom.

### 4.4 Further evaluation of the annotation quality and automating the annotation process

To assess the quality and consistency of the annotations in the “Personal Perspective” group, we train two transformer models to predict the sentiment in the response variables ‘feel’ and ‘surrender’ using Hugging Face ecosystem and related library (https://github.com/huggingface/transformers). The models are trained and tested on the annotations for ‘feel’ and ‘surrender’ responses performed by the three annotators. The purpose of this is two-fold. Firstly, we aim to evaluate the annotations by computing performance metrics such as accuracy and F1 score. The higher the values of these metrics, the more consistent and accurate the annotations are throughout the dataset. This evaluation of annotations is essential to ensure the quality of the data and the credibility of the results obtained from it. Secondly, we train these models to automatically predict the sentiments from user responses in future surveys, eliminating the need for manual annotation. This automation ensures the consistency and accuracy of future survey data while saving time and resources. By training these transformer models, we can improve the efficiency of the annotation process and obtain more reliable results in a shorter amount of time.

### 4.5 Estimating missing values

The dataset used in this study contains a significant proportion of null or missing values, as highlighted in Section 3. Specifically, out of the 455 observations, 313 contain one or more missing values, which represents about 69 % of the samples in the dataset. Simple univariate imputation methods like mean, maximum, or minimum values of the entire column or replacing null values with a new category may not perform well in survey data, as they do not consider other features to predict missing value in the current feature [26]. Hence, multivariate imputation methods that consider the correlations and relationships between variables are preferred [27].

Therefore, we used an iterative imputer based on random forest regression, which is a powerful imputation method for handling missing values in survey data [28]. This method estimates missing values by modeling each feature with missing values iteratively as a function of other features in the dataset, while considering correlations between features and using random forest regression to estimate the missing values. The iterative process continues until convergence, and the final imputed values are obtained by averaging the imputed values over multiple iterations.

Random forest regression is an effective imputation method because it can handle both continuous and categorical variables, and can also consider interactions between variables, which can be important in imputing missing values. Moreover, the iterative nature of the imputation process allows for better estimation of the imputed values over multiple iterations. Iterative imputation methods are better suited for survey data because they consider the relationships and correlations between variables in the dataset while estimating missing values. This method iteratively estimates missing values based on the current state of the dataset, allowing for better estimation of missing values while preserving the relationships between variables [28]. Thus, iterative imputation is considered a more robust method for handling missing data in survey datasets. For our dataset, we compare the performance of iterative imputer with other imputation methods such as zero-, (K Nearest Neighbors) KNN-, median-, mean-, and most-frequent imputation, and find iterative imputer to have the best performance in terms of root mean square error.

## 5 Analyzing sisu data through gradient boosting

Gradient boosting [29] is a machine learning technique that combines several weaker models (decision trees) into a single, strong predictor by iteratively training new models that focus on minimizing the errors of the previous models. These models learn from the errors (residuals) of their predecessors, with each new model focusing on the samples that the earlier models were unable to predict accurately. The final predictor is a weighted sum of all individual models containing the weights determined by their accuracy in predicting the target variable. Gradient boosting is a powerful method capable of dealing with nonlinear relationships and high-dimensional datasets [30]. It has shown impressive performance for various tasks such as image and speech recognition, natural language processing, and financial forecasting [[31], [32], [33], [34]].

### 5.1 Applying catboost for sisu data analysis

Catboost [35] is a machine learning framework which is built on some popular gradient boosting frameworks such as Extreme Gradient Boosting (XGBoost) and Light Gradient Boosting Machine (LightGBM), and incorporates several improvements over its predecessors, such as better handling of categorical features, automated feature scaling, and calculations of integrated feature importance. Catboost is designed to efficiently manage categorical features which makes it a suitable algorithm for datasets like sisu that contain a large number of categorical variables. Catboost can handle categorical features directly without requiring encoding. Catboost can convert categorical features into numerical values to enhance model accuracy while minimizing the risk of overfitting. Additionally, Catboost can automatically scale features, eliminating the need for manual feature scaling and reducing the risk of bias in the model. Another reason for selecting Catboost for sisu data analysis is its ability to handle missing values. The sisu dataset has a significant number of missing values, which can affect the performance of machine learning algorithms. Catboost's unique “Ordered Boosting” feature provides an efficient way of handling missing values in both numerical and categorical variables. Ordered Boosting treats missing values in categorical variables as a separate category and employs gradient-based optimization techniques to estimate missing data in numerical variables. To assess the performance of Catboost's missing value handling and our imputation method, we compare their accuracy and prediction error in our regression analysis. We find that both methods perform similarly in terms of accuracy and prediction error; however, the imputation method is slower than Catboost's missing value handling approach. Hence, we prefer to use Catboost's missing value handling feature for our regression analysis due to its speed and efficiency. In contrast, we find that our imputation method performs better than Catboost's missing value handling in classification analysis. Thus, we use our imputation method in the classification analysis to handle missing data.

### 5.2 Regression analysis

Regression analysis is a widely used statistical method in data analysis to explore the relationship between variables. It helps to identify the strength and direction of the relationship between a dependent variable and one or more independent variables. In the present study, we use regression analysis to examine the relationship between the selected predictors and target variables in the sisu dataset. Predictors are variables that could potentially influence the target variables, while the target variables are the variables we want to predict or understand better.

The regression analysis in our study aims to understand how the selected predictors influence the target variables. We considered three regression cases listed in Table 2, where we list predictors and targets for all cases. In all cases, we use demographic and employment variables along with personal perspective variables (Feel and Surrender) as predictors to understand how they influence the target variables of WHO5, Health status, and Excellent work performance.

**Table 2:** Predictors and targets in different cases of regression analysis.

Case	Predictor groups	Predictors	Target(s)
1	Demographics & Employment, Personal Perspectives group, sisu-sub factors	Age, gender, education level, surrender, feelings, sisu sub-factors	WHO5
2	Demographics & Employment, Personal Perspectives group, sisu-sub factors	Age, gender, education level, surrender, feelings, sisu sub-factors	Health status
3	Demographics & Employment, Personal Perspectives group, sisu-sub factors	Age, gender, education level, surrender, feelings, sisu sub-factors	Excellent work performance

In our study, we use the R-squared score to evaluate the performance of the regression models in predicting the target variables. The R-squared score gives us an indication of how well the independent variables explain the variation in the dependent variable. A higher R-squared score indicates a better fit of the regression model to the data.

It is important to note that both “health status” and “excellent work performance” are categorical variables. Yet, in our regression analysis, we have approached them as continuous variables. When classifying, we consider them categorical variables to further examine how each predictor impacts the various levels or classes of these variables.

### 5.3 Hyperparameter tuning

Hyperparameters are model parameters such as learning rate, regularization parameters, and the number of trees in gradient boosting models that cannot be determined from the data. The process of finding the optimal hyperparameters for training a machine learning model is called hyperparameter optimization. This optimization aims to find the best set of parameters that result in minimum prediction error (for regression analysis) or maximum accuracy (for classification) on the test dataset.

We use OPTUNA [36], an open-source hyperparameter optimization framework, to optimize the hyperparameters of our Catboost regression models. OPTUNA can explore the parameter search space (possible combination of parameter values) through different search techniques such as random search, Tree-Structured Parzen Estimator (TPE), and Covariance matrix adaptation evolution strategy (CMA-ES). The major advantage of OPTUNA is its search space exploration and convergence speed.

Initially, we define the search space for each hyperparameter, which includes its possible values and the distribution to sample from. Hyperparameter optimization is carried out in the form of multiple user-selected trials. In each trial, OPTUNA selects a set of hyperparameters based on the sampling strategy which determines the method of exploring the hyperparameter search space for the optimization task. OPTUNA offers a number of sampling strategies to navigate the search space, including random search, grid search, and several forms of Bayesian optimization. The number of trials is specified by the user, and each trial is performed iteratively to minimize/maximize an objective function. In our study, we formulate the OPTUNA objective of maximizing the R-square value. Hence, OPTUNA follows an optimization direction that maximizes the R-square values, which in turn indicates the goodness of fit of the regression model.

**Fig. 6:**
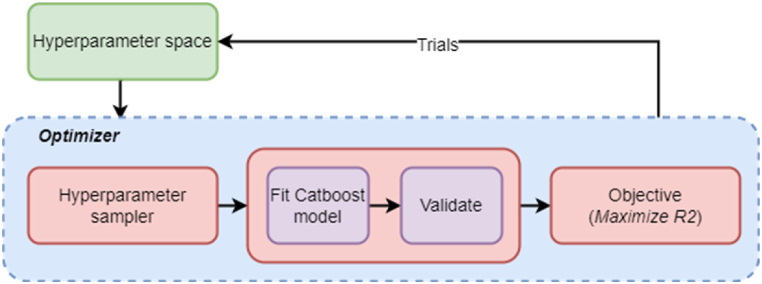
Hyperparameter tuning.

Due to the variability of the sisu dataset, we use *K*-fold cross-validation during each trial to fit the model and obtain stable results. Cross-validation is a widely used technique for assessing the performance of machine learning models and estimating their generalization error. In our study, we use the 5-fold cross-validation technique to split the data into 5 subsets of equal size, train the model on four subsets, and evaluate its performance on the remaining subset. This process is repeated five times, with each subset being used once as the validation set. The results obtained from the five folds are then averaged to obtain a stable estimate of the model's performance. Fig. 6 depicts the process of hyperparameter tuning. Table 3, Table 4 show the hyperparameters considered for tuning for regression and classification scenarios.

**Table 3:** Hyperparameters used for tuning for the regression scenario.

Parameter	Range/Values
Number of Boosting Steps	100 to 1000
Loss Function	“MultiRMSE"
Learning Rate	0.001 to 0.5 (log-uniform distribution)
L2 Regularization	0.01 to 1.0 (log-uniform distribution)
Feature Sampling Ratio	0.01 to 0.1
Tree Depth	1 to 10
Boosting Type	“Ordered”, “Plain"
Bootstrap Method	“Bayesian”, “Bernoulli”, “MVS"
Minimum Data in Leaf	2 to 20
Max Categories for One-Hot	2 to 20

**Table 4:** Hyperparameters used for tuning for the classification scenario.

Parameter Description	Range/Values
Number of Boosting Steps	10 to 1000
Learning Rate	0.001 to 0.3 (log-uniform distribution)
Tree Depth	1 to 10
L2 Regularization	1e-8 to 10.0 (log-uniform distribution)
Randomness of Boosting	1e-8 to 10.0 (log-uniform distribution)
Bagging Temperature	1e-8 to 10.0 (log-uniform distribution)
Random Subspace Method	0.0 to 1.0 (uniform distribution)
Loss Function	“Logloss”, “CrossEntropy"
Border Count	1 to 255
Boosting Type	“Plain”, “Ordered"
Leaf Estimation Method	“Newton”, “Gradient"
Feature Border Selection	“MinEntropy”, “GreedyLogSum"

### 5.4 Classification analysis

Classification analysis is an essential machine learning method in data analysis, where we use it to classify observations into different categories or classes. Unlike regression analysis, where the target variable is continuous, classification analysis predicts a categorical target variable. In our study, we use classification analysis to examine the relationship between the selected predictors and target variables in the sisu dataset.

In the sisu data analysis, we use classification analysis to predict the target variables such as “surrender”, “feelings”, “overall life satisfaction”, “health status”, and “excellent work performance” based on the selected predictors. The selected predictors in the classification analysis include demographic and employment variables, personal perspective variables, and sisu sub-factors. To evaluate the performance of the classification models, we use accuracy, precision, recall, and F1 score. Higher accuracy, precision, recall, and F1 score indicate better classification performance of the model.

The hyperparameter tuning is done using OPTUNA in the same way as described in Section 5.3. The only difference is that the objective of optimization in our classification cases is to maximize accuracy. Table 5 summarizes the predictors and targets used in the classification analysis.

**Table 5:** Predictors and targets in different cases of classification analysis.

Case	Predictors	Target
1–2	Demographics & Employment Group (age, gender, education level), sisu sub-factors	Surrender
		Feelings
3–5	Demographics & Employment Group (age, gender, education level), Personal perspective group (surrender, feel), sisu sub-factors	Overall life satisfaction
		Health status
		Excellent work performance

### 5.5 Explainable artificial intelligence using SHAP method

SHAP (SHapley Additive exPlanations) is a unified framework for interpreting machine learning model predictions, grounded in cooperative game theory [37]. It assigns a Shapley value to each data feature, quantifying its contribution to a particular prediction. In the context of both regression and classification models, SHAP values offer granular insights into the impact of each feature on the model's output, thereby facilitating a more transparent and interpretable model. The motivation for using SHAP in the realm of explainable artificial intelligence stems from its ability to provide both local and global explanations, making it versatile for various applications. Local explanations help in understanding individual predictions, while global explanations offer an overview of the model's behavior across the dataset. Explainability of predictions is necessary here as we aim to uncover how a wide range of human behavior and psychological traits affect sisu and vice versa. SHAP's solid mathematical foundation ensures that the explanations are consistent and fairly distributed among the features (see e.g. Ref. [38]).

## 6 Results

This section presents the results of regression and classification analysis.

### 6.1 Clustering of perspective group variables

**Fig. 7:**
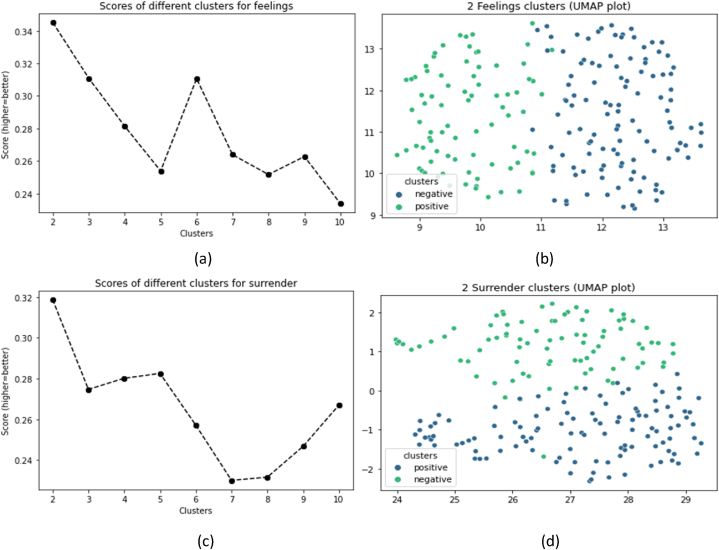
Silhouette scores of different number of clusters (a, c), and optimal clusters (b,d).

Ten different models are created using the number of clusters ranging from 2 to 10. Once each number of clusters is applied to K-means clustering, the Silhouette score is calculated. The Silhouette score profile for all clusters is displayed in Fig. 7a, c. The highest score denotes the ideal number of clusters, which is 2 in both cases.

To understand the nature of these clusters, we conduct a thorough examination of the textual responses aggregated within each cluster. For both textual responses, the first cluster contains responses that predominantly expressed a positive or resilient sentiment. Whereas the other second cluster comprises responses that reflected a more negative or defeatist sentiment. Hence, these clusters essentially represent two polarized emotional responses and coping strategies extracted from the “feel” and “surrender” variables.

b, d presents the UMAP representation of the optimal number of clusters, demonstrating K-means' ability to obtain a clear separation of clusters. The text embeddings were reduced to a 5-dimensional space. However, the scatter plots (Fig. 7) display data in 2 dimensions for better interpretation and visualization. These two dimensions are new axes calculated to preserve, as much as possible, the high-dimensional distances after projection into 2D space.

### 6.2 Annotation and classification of perspective group variables

After annotating the “surrender” and “feel” responses as negative (0) and positive (1) in accordance with the method described in Section 4.2, the inter-rater variability between the three annotators was evaluated by calculating Cohen's Kappa (as explained in Section 4.3). These coefficients, along with their respective confidence intervals, suggest a good level of agreement between annotator pairs in their categorization of the responses. This indicates that the categorization of the responses as negative or positive was done consistently by both annotators, and their ratings can be considered reliable. The results of the inter-rater variability and sentiment analysis are reported in Table 6. Finally, majority voting is used for the final annotations. The annotators referred to in the column ‘Annotator Pair’ are the first author (1), second author (2) and a research assistant (3).

**Table 6:** Kappa coefficients and confidence intervals of the perspective group variables.

Variable	Annotator Pair	Kappa	Binomial Test Ratio	Binomial *P*-Value	McNemar *P*-Value	Classification Accuracy	F1
Surrender	(1, 2)	0.850629	0.934211	5.568238e-46	1.184692e-01	0.96	0.97
	(1, 3)	0.682477	0.846491	1.175192e-27	2.095476e-09		
	(2, 3)	0.584995	0.798246	2.497825e-20	1.564172e-04		
	**Avg.**	**0.706033**					
Feelings	(1, 2)	0.858841	0.930909	3.442624e-54	4.425049e-03	0.97	0.97
	(1, 3)	0.785555	0.894545	5.026774e-44	2.649309e-01		
	(2, 3)	0.789790	0.898182	5.872123e-45	3.449285e-01		
	**Avg.**	**0.811395**					

The high kappa values in Table 6 indicate a significant agreement among annotators, confirming the reliability of sentiment classifications for ‘feel’ and ‘surrender.’ Near-unanimous binomial test ratios further underscore this consistency, suggesting a common understanding of sentiment definitions. The F1 scores, approaching 1, reflect high precision and recall, indicating a balanced, unbiased representation of sentiments. Moreover, the extremely low binomial and McNemar p-values affirm that the agreement is statistically significant, not just random coincidence.

We tokenize the training and test data for both the target variables, i.e., “feel” and “surrender”, followed by loading the transformer model from Hugging Face model library^2^, ^3^. Then, we fine-tune the transformer models for the respective target variables by retraining them using the training dataset. The training arguments for the model are set up, including the learning rate of 2e^−5^, per device batch size of 16, two training epochs, and weight decay of 0.01. The trainer object is then set up with the fine-tuned model and training arguments, and the model's performance is evaluated on the test dataset. The evaluation metrics include accuracy and F1 score.

### 6.3 Regression analysis

Table 7 shows the results of the 3 cases of regression analysis. It is common to have a small R-squared value in survey data, including responses from the participants. This is due to the fact that survey data often includes a high degree of variability, and the predictors may not fully capture all the factors that affect the target variable. Additionally, survey data may include measurement errors, and some participants may provide inconsistent or unreliable responses. These factors can contribute to a lower R-squared value and make it challenging to accurately predict the target variable.

**Table 7:** Results of regression analysis.

Predictors	Targets	R2
Age, gender, education level, surrender, feelings, sisu sub-factors	WHO5	0.2972
	Health status	0.1370
	Excellent work performance	0.1048

The model output across all observations in the dataset. The red points on the graph indicate higher values of the data, while the blue points represent smaller data values.

**Fig. 8:**
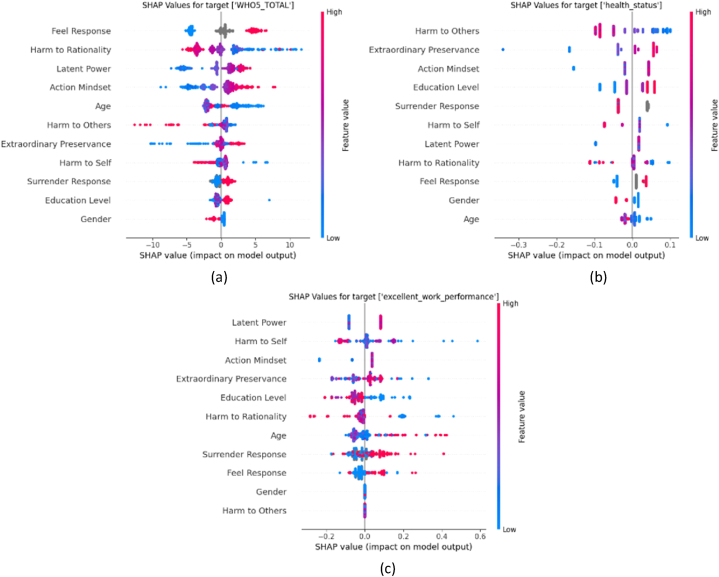
Beeswarm plots of (a) WHO5, (b) health status, and (c) excellent work performance.

The beeswarm plots showing the SHAP values of each predictor for WHO5, health status, and excellent work performance are depicted in Fig. 8. The SHAP values represent the impact of each predictor to.

The vertical line in the middle of the graph represents the zero SHAP value, which means that the corresponding feature has no effect on the target variable. The points on the right side of the graph represent a positive outcome on the prediction, which means a higher predicted value. For instance, in the case of predicting WHO5 (Fig. 8a), the “feel” response has a positive correlation with WHO5. The red SHAP values on the right side of the plot indicate the higher-valued responses (“feel” = 1) which are associated with higher values of WHO5, indicating that the “feel-good” responses are linked to higher wellbeing.

### 6.4 Classification analysis

Table 8 shows the results of the classification analysis performed on the sisu dataset. We train a baseline classifier using the “most frequent” strategy suitable for imbalanced datasets [39] and employ 5-fold cross-validation to establish a stable performance level for each classification case. The performance metrics of the target variables “overall life satisfaction”, “health status” and “excellent work performance” do not depict an impressive prediction performance; however, our main aim is not to achieve a higher performance metrics, but to gain insights into how the individual predictors affect the target variables.

**Table 8:** Results of classification analysis.

Predictors	Targets	Accuracy	Precision	Recall	F1 score
Age, gender, education level, sisu sub-factors	Surrender	0.8461 (Baseline: 0.7877)	0.8529	0.8461	0.8491
	Feelings	0.7384 (Baseline: 0.6050)	0.6691	0.7384	0.6993
Age, gender, education level, surrender, feelings, sisu sub-factors	Overall life satisfaction	0.5648 (Baseline: 0.5604)	0.7481	0.5648	0.6416
	Health status	0.4747 (Baseline: 0.4527)	0.5660	0.4747	0.5139
	Excellent work performance	0.5670 (Baseline: 0.5212)	0.5702	0.5670	0.5664

**Fig. 9:**
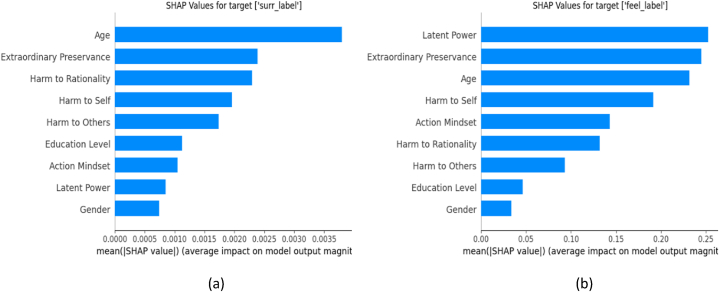
SHAP summary graphs of (a) “surrender”, and (b) “feel” variables.

**Fig. 10:**
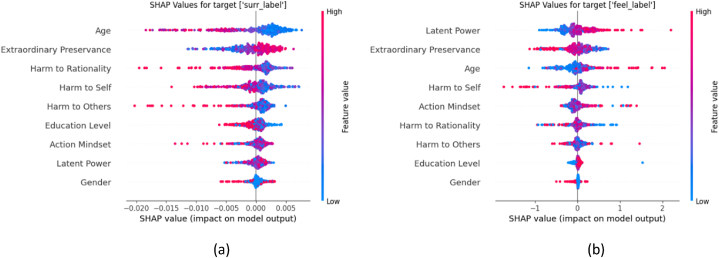
Beeswarm plots of (a) ‘surrender’, and (b) ‘feel’ variables.

Fig. 9 illustrates the mean SHAP values of each predictor for both “surrender” and “feel” labels. Subsequently, Fig. 10 provides beeswarm plots, visually representing individual SHAP values of every predictor for both labels. In the process of interpreting a CatBoost model with SHAP, the model initially generates predictions in log-odds for every instance within the dataset. SHAP then calculates values based on these log-odds. Notably, the summation of the model's expected value—representing the average output of the model (in log-odds) across the entire dataset—and the SHAP values for each feature within a specific instance will equate to that instance's log-odd output. For enhanced interpretability, these log-odd values are then transformed into probabilities using the logistic function. In Fig. 10, the SHAP values signify the extent and direction of a feature's impact on the prediction when compared to the model's expected value. In binary classification terms, a positive SHAP value pushes the prediction towards class 1, while a negative one pushes it towards class 0.

**Fig. 11:**
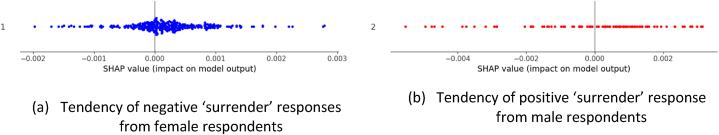
Differential influence of gender categories on ‘surrender’ variable.

**Fig. 12:**
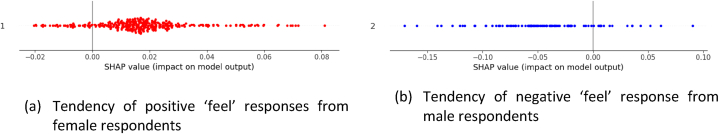
Differential influence of gender categories on ‘feel variable.

It is evident that age and latent power stand out as the most dominating factors for surrender and feel labels, respectively. We analyze the SHAP values distribution of individual categories. For example, the aggregated distribution of SHAP values for “gender” variable, as shown in Fig. 10a, may not provide a visually comprehensive insight. To examine the relative significance of categories within the “gender” feature, we calculate the mean SHAP values for each category and compare them with the overall mean SHAP values. We assign colors to the categories based on their mean SHAP values. If the mean SHAP value of a category is higher than the overall mean, it is assigned red color, which shows a higher concentration of the values of this category on positive side. Conversely, if the mean SHAP value is lower than the overall mean, the category is assigned blue color, representing a higher concentration of the values of this category on negative side. The beeswarm plots, shown in Fig. 11a, b and Fig. 12a,b, provide a granular perspective of how each category of “surrender” and “feel” responses influences the model's overall predictions. In Fig. 11a, the graph representing category 1 (female) is assigned a blue color which indicates that the answers of “surrender” question by female respondents tend to have lower SHAP values (negative response). On the other hand, the graphs for category 2 (male) in Fig. 11b are assigned red color which represents a tendency of positive response. Similarly, Fig. 12a, b shows a tendency of positive response for “feel” variable by female respondents.

**Fig. 13:**
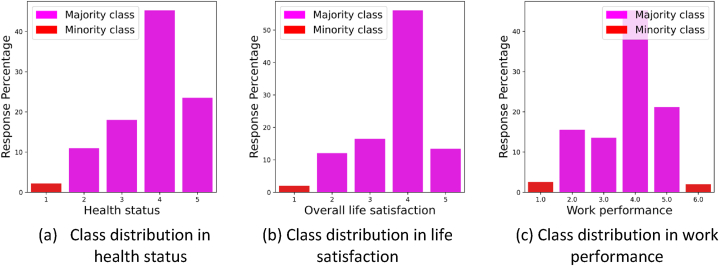
Minority and majority classes in each variable.

The other target variables – “overall life satisfaction”, “health status”, and “excellent work performance” - each contain multiple classes. Our analysis extends to the detailed examination of the beeswarm plots for each individual class. However, we do consider that each variable contains classes that are underrepresented. Thus, to get precise insights, we focus primarily on the majority classes when assessing the beeswarm plots. Fig. 13 shows the minority and majority classes in each variable. For instance, in the context of “overall life satisfaction”, we examine beeswarm plot of class 2 to find the influence of the variables in lower level of life satisfaction, as class 1 is underrepresented. Fig. 13 further illustrates that the underrepresented classes are positioned at the extremes, representing a smaller number of respondents at these levels. The figure highlights that although the number of respondents experiencing the most challenging conditions is relatively low, the representation of those achieving the ideal work performance (level 6) is also sparse.

**Fig. 14:**
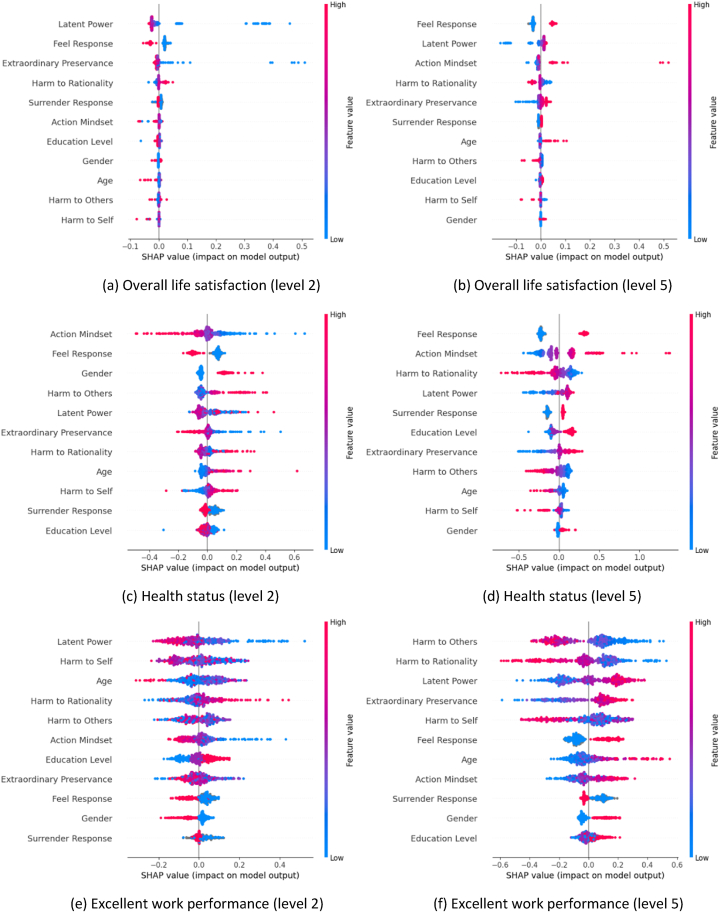
Beeswarm plots of overall life satisfaction (a,b), health status (c,d), and excellent work performance (e,f).

Fig. 14 shows the beeswarm plots for the classes at both ends of the spectrum for each target variable. In these plots, the lower level is indicative of suboptimal conditions, whereas the higher level signifies prosperous conditions.

## 7 Discussion

When we explore the intricate web of relationships and influence dynamics between sisu sub-factors and other variables through the lens of explainable AI, we uncover a plethora of intriguing insights. We employ a multidisciplinary theoretical framework that integrates psychological constructs of sisu with machine learning methodologies, specifically Explainable AI (XAI). At the core is the Sisu Scale, a validated instrument that measures both beneficial and harmful aspects of sisu. Using this scale, we examine the complex interplay between sisu and various factors such as wellbeing, work stress, and health status. XAIprovided us nuanced understanding of relationships and dynamics among the variables that goes beyond traditional statistical analyses. The regression and classification analysis, albeit showing appreciable predictability, serves as a valuable tool for scrutinizing these interconnections. Next, we discuss how sisu corresponds to health, well-being, work performance and written responses.

### 7.1 Beneficial sisu enhances well-being and health while harmful sisu undermines them

In relation to WHO5 wellbeing (Fig. 8a), the sub-factors of beneficial sisu exert a favorable impact on the target, while the harmful sisu sub-factors tend to adversely affect the target variable. This is because the positive traits associated with sisu enhance overall well-being, whereas negative traits may cause stress or dissatisfaction [7]. Interestingly, a younger demographic seems to positively correlate with WHO5. This is because younger individuals are more adaptive and flexible to changing circumstances, which could result in better overall wellbeing scores.

Furthermore, the perspective variables (“feel” and “surrender”), along with higher education levels, appear to positively influence WHO5. This has also been confirmed by a study which states that higher education levels have been shown to lead to a higher level of wellbeing [40]. While gender seems to play a minor role in influencing WHO5, the results show a slight positive influence on females. It is critical to note, though, that this observation does not necessarily indicate a causal relationship due to the least dominating role of gender variable. Further exploration may be needed to understand the underlying mechanisms.

In the analysis of health status (as depicted in Fig. 8b), we find that education level, feel, and beneficial sisu factors generally have a positive influence on health status. This supports the notion that higher education levels and more positive mindsets contribute to better health outcomes [40]. Conversely, the harmful sisu factors appear to negatively affect health status. This suggests that respondents with higher beneficial sisu and lower harmful sisu experience better health status. The vertical alignment of SHAP values observed in this plot signifies that a significant portion of these variables' values share identical or very similar SHAP values, resulting in the formation of these vertical lines.

### 7.2 Beneficial sisu and positive outlook boost work performance

With regards to work performance (Fig. 8c), we observe that higher values of beneficial sisu sub-factors have a more substantial role in enhancing work performance. This confirms that the individuals who display these positive qualities, such as resilience and perseverance, may be better equipped to excel in their professional pursuits [41]. The perspective variables “feel”, and “surrender” show a consistent positive influence on the model's output prediction. This means that respondents who maintain a positive outlook and show flexibility in their attitudes are more likely to perform better at work.

The education level is negatively correlated with work performance. One potential explanation could be that higher education might instill more theoretical knowledge which may not always translate into practical skills required for efficient work performance. However, research shows that the correlation between education level and job performance is weak, and that intelligence scores are a much better indicator of job potential [42]. The gender factor appears to be insignificant in this context, suggesting that work performance is not influenced by gender. This is also supported by the study that suggests that gender only has minimal effects on work performance [43].

Fig. 14e–f shows that the influence of beneficial sisu shifts from being less prominent at lower levels to a more substantial impact at higher performance levels. Factors such as advanced age and higher education level contribute positively to work performance. This correlation can be attributed to the accumulation of experience, skills, and knowledge over time, leading to improved work performance. The ‘feel’ variable presents an interesting dynamic – it transitions from exerting a negative influence on work performance at lower levels to a positive one at higher levels, signifying the impact of feelings on the performance level. Gender plays a relatively minor role in work performance. However, male respondents tend to have better work performance. This could be attributed to the societal or workplace dynamics, cultural conditioning, or personal attributes associated with these gender categories.

### 7.3 Younger age and beneficial sisu boost mental resilience

The role of age in predicting responses to the “surrender” (mental resilience) question is most dominant (Fig. 9a), with younger individuals more likely to respond positively (Fig. 10a). This indicates that younger people, often more adaptable and open to learning, are more willing to re-evaluate their goals and change course, if necessary [44], a characteristic that could be seen as a form of resilience or flexibility.

The individuals with lower levels of harmful sisu factors are also more likely to respond positively to the “surrender” question. This suggests that lower levels of hardships or challenges could potentially result in a more optimistic or resilient attitude when faced with situations that might require surrender. Conversely, higher levels of beneficial sisu factors also correspond with positive responses, which might suggest a correlation between a resilient, positive attitude and the ability to adapt or surrender when appropriate.

While gender plays a less dominant role in the analysis (Fig. 10a), the tendency for males to respond positively to the “surrender” question is indicative of societal expectations and norms around masculinity, such as the belief that being resilient and able to adapt to changing circumstances is a sign of strength. It is interesting to note that the individuals with a higher education level tend to provide negative responses for the “surrender” question (Fig. 10a). This suggests that these respondents, who are typically well-equipped with knowledge and problem-solving skills, can lean towards a more analytical and practical approach when dealing with complex issues. This manifests as an inclination to explore all possibilities and consider the potential for failure, which might be interpreted as “surrendering” in certain situations.

7.4 Higher Perseverance, Higher Latent Power, Female Gender, and Higher Education Positively Affect Feelings.

The responses to the “feel” variable (see Fig. 9, Fig. 10b) offer intriguing insights into the relationship between sisu factors, gender, and education level. Extraordinary perseverance, a beneficial sisu factor, appears to have a substantial influence on the responses, suggesting that individuals with a high perseverance are more inclined to respond positively to questions about their feelings. This stems from their enhanced capacity for resilience and perseverance, traits inherent in the sisu concept which contribute to a more optimistic or positive emotional state. Further, individuals with high latent power seem to respond positively to the “feel” question more frequently. This suggests that high latent power in the face of adversity or challenge have a greater sense of emotional well-being or positivity. Gender also seems to play a role in responses, with females tending to give positive responses more frequently (see Fig. 10b). This is reflective of societal expectations, where women are often encouraged to be more open about their feelings [45]. This results in a higher likelihood of females acknowledging or expressing positive feelings.

Respondents with a higher education level appear to give a positive response to the “feel” variable more often (see Fig. 10b). This is confirmed by the recent studies which state that a higher education level is found to be linked with improved health and mental well-being, leading to a positive emotional state [45,46].

### 7.4 Positive “feel”, beneficial sisu, higher education, and higher mental resilience enhance life satisfaction

When considering “overall life satisfaction” (Fig. 14a–b), the variable “feel” emerges as an influential factor. The results show that lesser life satisfaction often coincides with negative emotional states. It further strengthens the notion that respondents with reduced beneficial sisu factors and increased harmful sisu sub-factors tend to experience lower levels of life satisfaction. However, the beeswarm plot for the highest life satisfaction level (Fig. 14b) paints an entirely different portrait. It is evident that positive “feel” responses, higher values of beneficial sisu sub-factors, higher educational level, higher age, and higher mental resilience (represented through positive “surrender” responses) all correlate with superior life satisfaction levels. While gender appears to play a relatively small part in determining the highest levels of life satisfaction, it is notable that male respondents tend to be positively associated with greater life satisfaction. However, the minimal influence of gender variable on the highest levels of life satisfaction keeps us inferring broad generalizations from this observation.

Emotional state, represented by “feel” variable, significantly impacts respondents' “overall life satisfaction.” (See Fig. 14a–b). High levels of negative emotions are associated with lesser life satisfaction. This connection stems from the fact that emotional distress can negatively affect one's outlook on life and overall well-being [47,48]. Additionally, a lower level of beneficial sisu traits, combined with a higher level of harmful sisu traits, tends to coincide with diminished life satisfaction. This correlation could be attributed to the role that resilience and perseverance play in overcoming life's obstacles. Individuals with higher resilience can better manage life's challenges, resulting in increased satisfaction with life. On the flip side, positive emotional responses, stronger beneficial sisu traits, higher education levels, advanced age, and greater mental resilience (indicated by positive “surrender” responses) all seem to boost life satisfaction. This is because these factors collectively contribute to a sense of achievement, self-worth, and capacity to handle life's ups and downs, which are all crucial elements in overall life satisfaction. While gender appears to have a limited role in determining life satisfaction levels, male respondents show a slightly positive association with higher life satisfaction. This observation could potentially be tied to societal or cultural factors, including different expectations or societal roles based on gender.

### 7.5 Positive “feel”, beneficial sisu, higher mental resilience, younger age, and higher education boost health status

Exploring the “health status” deeper, it is notable how the emotional state, as represented by the “feel” variable, plays a dominant role (see Fig. 14c–d). It is evident from our analysis that several factors contribute collectively to a higher health status. These include a positive emotional state, increased beneficial sisu traits, decreased harmful sisu traits, strong mental resilience indicated by positive “surrender” responses, younger age, and higher education level. This suggests that maintaining a positive mindset, enhancing beneficial sisu traits while reducing negative ones, demonstrating mental resilience, and investing in education are beneficial to an individual's health status. These findings highlight the importance of psychological traits and emotional well-being in maintaining good health. As for the role of gender, it appears to have a minor influence on good health status (see Fig. 14d). However, our analysis shows that female respondents seem to have a slightly negative effect on their overall health status. This could potentially point towards gender-specific health issues or societal pressures impacting women's health.

## 8 Conclusion & future work

This study employed the concepts of explainable AI to explore the interrelations between various factors like sisu, age, gender, education, feelings, health status, and work performance. We adopted a multidisciplinary theoretical framework that merges the psychological concept of sisu, specifically the Sisu Scale, with machine learning. We applied explainable AI in the analysis of complex relationships between sisu and variables such as life satisfaction, work performance, and health status. This integration aims to uncover predictive mechanisms and inform intervention design to advance the understanding of sisu's dual impact on individual and societal well-being. Our approach enabled us to gain insightful correlations and trends hidden within the complexity of the dataset. Our study unveiled correlations and relationships between a wide array of influencing factors, thereby setting our work apart from previous research in this area.

The analysis revealed that beneficial sisu factors significantly enhance life satisfaction and health status, whereas their negative counterparts tend to lower these metrics. This emphasizes the crucial role sisu plays in an individual's wellbeing and lifestyle. Our investigation uncovered the effect of subjective feelings on life satisfaction, health status, and work performance. Positive emotional states were frequently associated with improved outcomes in these areas, highlighting the connection between emotional well-being and overall life quality.

Gender also had a minor effect on certain trends, such as male respondents experiencing slightly higher work performance and life satisfaction. Regarding the open-text responses, the “surrender” question in particular suggests that mental resilience, as indicated by positive responses, is positively associated with life satisfaction, health status, and work performance. Our results confirm the previous findings and our hypothesis that beneficial sisu is on average associated with higher levels of well-being, better health status, enhanced work performance, and increased life satisfaction, while the opposite holds for harmful sisu.

We recognize a limitation of this study being the significant proportion of missing values and a relatively smaller number of observations. Additionally, there exists an imbalance in the gender class. A more comprehensive and balanced dataset might yield deeper insights into the interplay of sisu factors.

Overall, this study has illustrated the subtle dynamics between various aspects of life through the lens of explainable AI which demonstrates the potential of this approach for uncovering critical insights that can be applied in fields like psychology, sociology, and health sciences, among others. Future research may seek to extend these findings to more diverse datasets and contexts, as well as an in-depth analysis of the patterns and trends uncovered in this study.

## Author contributions

Pentti Henttonen: Writing – review & editing, Writing – original draft, Funding acquisition, Data curation. Ilmari Määttänen: Writing – review & editing, Writing – original draft, Funding acquisition, Data curation. Umair Ali Khan: Writing – review & editing, Writing – original draft, Validation, Methodology, Investigation, Formal analysis, Conceptualization. Janne Kauttonen: Writing – review & editing, Writing – original draft, Formal analysis

## Data Availability Statement

The data that has been used is confidential and is not deposited to any publicly available repository.

## Declaration of competing interest

The authors declare the following financial interests/personal relationships which may be considered as potential competing interests: Henttonen Pentti reports financial support was provided by European Social Funds Plus. If there are other authors, they declare that they have no known competing financial interests or personal relationships that could have appeared to influence the work reported in this paper.

## Footnotes

1 Kyselyn Palaute.

2 https://huggingface.co/umairalipathan/finetuning-sentiment-model-feeling-final.

3 https://huggingface.co/umairalipathan/finetuning-sentiment-model-surrender-final.
